# Glucose transporter 3 (GLUT3) promotes lactylation modifications by regulating lactate dehydrogenase A (LDHA) in gastric cancer

**DOI:** 10.1186/s12935-023-03162-8

**Published:** 2023-12-01

**Authors:** Hao Yang, Shifeng Yang, Jixing He, Wenqiang Li, Ange Zhang, Nana Li, Guangkai Zhou, Boshi Sun

**Affiliations:** 1 Department of General Surgery, The Second Affiliated Hospital of Harbin Medical University, Harbin, Heilongjiang, China; 2 Department of Head and Neck Surgery, Harbin Medical University Cancer Hospital, Harbin, China; 3 Department of General Surgery, Jinshan Hospital of Fudan University, Shanghai, China

**Keywords:** Glucose transporter 3, Lactylation, Lactate dehydrogenase A, Epithelial-mesenchymal transition, Gastric cancer

## Abstract

**Objectives:**

Glucose transporter 3 (GLUT3) plays a major role in glycolysis and glucose metabolism in cancer cells. We aimed to investigate the correlation between GLUT3 and histone lactylation modification in the occurrence and progression of gastric cancer.

**Materials and methods:**

We initially used single-cell sequencing data to determine the expression levels of *GLUT3* and lactate dehydrogenase A (LDHA) in primary tumor, tumor-adjacent normal, and metastasis tumor tissues. Immunohistochemistry analysis was conducted to measure GLUT3, LDHA, and L-lactyl levels in gastric normal and cancer tissues. Transwell and scratch assays were performed to evaluate the metastatic and invasive capacity of gastric cancer cell lines. Western blotting was used to measure L-lactyl and histone lactylation levels in gastric cancer cell lines.

**Results:**

Single-cell sequencing data showed that *GLUT3* expression was significantly increased in primary tumor and metastasis tumor tissues. In addition, *GLUT3* expression was positively correlated with that of *LDHA* expression and lactylation-related pathways. Western blotting and immunohistochemistry analyses revealed that GLUT3 was highly expressed in gastric cancer tissues and cell lines. *GLUT3* knockdown in gastric cancer cell lines inhibited their metastatic and invasive capacity to various degrees. Additionally, the levels of LDHA, L-lactyl, H3K9, H3K18, and H3K56 significantly decreased after *GLUT3* knockdown, indicating that GLUT3 affects lactylation in gastric cancer cells. Moreover, *LDHA* overexpression in a *GLUT3* knockdown cell line reversed the levels of lactylation and EMT-related markers, and the EMT functional phenotype induced by *GLUT3* knockdown. The in vivo results were consistent with the in vitro results.

**Conclusions:**

This study suggests the important role of histone lactylation in the occurrence and progression of gastric cancer, and GLUT3 may be a new diagnostic marker and therapeutic target for gastric cancer.

**Supplementary Information:**

The online version contains supplementary material available at 10.1186/s12935-023-03162-8.

## Introduction

Gastric cancer (GC), a cancer of the upper gastrointestinal (GI) tract that has a poor prognosis and presents a serious threat to human health. The burden of this disease in China is severe, as the number of new cases of gastric cancer accounts for 44% of cases worldwide [1]. Altered energy metabolism is a major hallmark of gastric cancer and is a key mechanism endowing gastric cancer cells with invasion and metastasis abilities [2]. Metabolic reprogramming characterized by increased glycolysis is associated with multiple steps in the development and progression of gastric cancer [3].

Lactate is a metabolic product of glycolysis that was initially considered a metabolic waste product. However, studies have shown that lactate has various important physiological and pathological functions. In particular, it can lead to the lactylation of histone lysine residues in macrophages to regulate gene transcription [4–7] Lactate dehydrogenase A (LDHA) is a key enzyme in glycolysis that is highly expressed in various GI tumors, including gastric, esophageal, and pancreatic cancers. It is often associated with poor prognosis and high rates of metastasis [8]. Increases in LDHA levels in gastric cancer can increase lactylation levels [9]. However, the specific mechanisms and pathways responsible for the increases in *LDHA* expression and lactylation in gastric cancer remain unknown.

Glucose is the most important energy source for tumor cells, providing energy for their growth and metabolism. Glucose molecules are highly hydrophilic and cannot freely pass through hydrophobic biological membranes. Therefore, cell membrane transporter proteins are necessary for their entry and exit from tumor cells. Glucose transporters (GLUTs) are members of the major facilitator superfamily that are essential to this process. Of the 14 GLUTs known in humans, GLUT1, GLUT2, GLUT3, and GLUT4 are responsible for transporting glucose to different tissues in the body [10]. Numerous recent studies have shown that *GLUT3* is overexpressed in various solid tumors owing to the rapid proliferation of tumor cells, which results in the formation of a hypoxic environment. Moreover, some studies have shown that GLUT3 has a greater influence on cancer cell growth than GLUT1 in conditions of glucose deficiency [11]. Moreover, GLUT3 was found to be significantly correlated with poor prognosis in gastric cancer, while other members of the GLUT family were not associated with prognosis [12]. Therfore, we chose GLUT3 as the main object of our study.

Thus, tumor cells rely on glycolysis under anaerobic conditions and produce over 10 times less adenosine triphosphate (ATP) than under aerobic conditions [13]. The energy demand of patients with gastric cancer is much higher than that of healthy individuals; approximately 26% of patients with gastric cancer are in a hypermetabolic state, with an energy consumption of over 110% at rest. Glucose consumption is significantly higher in patients with gastric cancer at advanced stages. Moreover, the increase in the glucose oxidation rate is proportional to the size of the tumor [14] Gastric cancer cells must express GLUTs at high levels to achieve a high glucose intake and energy metabolism [15]. A large amount of lactate accumulates in gastric cancer cells with increased glucose metabolism and glycolysis. In turn, excess lactate leads to lactylation of histones, which promotes gastric cancer progression and invasiveness.Therefore, the in-depth study of the relationship amon GLUT3, glucose metabolism, and lactylation would be valuable for predicting gastric cancer prognosis and providing guidance for treatment. In this study, LDHA was used as the key regulatory target of lactylation, and it was revealed that the expression level of LDHA was proportional to the degree of lactylation in gastric cancer. This is the first study to correlate GLUT3 and LDHA with lactatylation, which could be important for guiding subsequent studies on lactylation in gastric cancer.

## Materials and methods

### Single-cell data download and processing

Single-cell data of gastric cancer were downloaded from the Gene Expression.

Omnibus (GEO) database (GSE183904; https://www.ncbi.nlm.nih.gov/geo/) [16]. This dataset contains results from 40 gastric cancers, including 29 tumor samples (26 in situ tumors and 3 peritoneal metastatic tumors) and 11 samples of healthy tumor-adjacent tissue (10 in situ controls and 1 peritoneal metastatic control). All samples were selected for subsequent analyses.

Analysis of the entire single-cell transcriptome was performed using the R Seurat package (version 4.0.3). The expression matrices from different sample sources were renamed for data merging. Quality control of data was performed by removing low-quality cells, cell fragments, and possible double cells. Cells with a gene count of 500–6000 and mitochondrial gene percentage of 20% or less were selected for subsequent analysis. This resulted in a total of 158,500 entries of high-quality single-cell expression data.

### Comprehensive analysis of single-cell data and cell type annotation

High-quality expression data of 40 samples were obtained after data preprocessing and quality control. To avoid batch effects, the canonical correlation analysis algorithm was used for unbiased integration of multiple samples. First, the SplitObject function was used for array partition based on sample groups. Subsequently, the NormalizeData function was used to standardize the data. Consequently, the raw expression values were converted to standardized and comparable data by removal of the effects of sequencing depth. The FindVariableFeatures function was used (with 2000 highly variable genes selected by default) to calculate highly variable genes for each sample. The FindIntegrationAnchors function was used to find similarity anchors between the two pairs of data. Finally, the IntegrateData function was used to integrate multiple sample groups. Clustering and dimensionality reduction of data, and cell type annotation were performed after sample integration. Subpopulations without signature gene expression and subpopulations expressing two or more signature genes were defined as low-quality cells and double cells, respectively. They were uniformly removed from the dataset, without subsequent analysis. Finally, a total of 151,874 entries of high-quality single-cell expression data remained.

### Single-cell status scoring

A single-cell scoring algorithm was used to evaluate the status of individual cells in a specific set of functional genes or pathways. Scoring of single cells was performed using the AddModuleScore function in Seurat. The used pathways and genes were downloaded from the Molecular Signatures Database (v7.5.1) (https://www.gsea-msigdb.org/gsea/index.jsp).

### Cell type subpopulations

To subclassify an annotated cell type, all cells of that cell type were extracted using the subset function. Subsequently, the extracted cells were integrated, subclustered, and annotated using the same process as in the “Comprehensive analysis of single-cell data and cell type annotation” section.

### Visualization of single-cell data

The following functions or R packages were used for visualization of single-cell data. (1) DimPlot: Uniform Manifold Approximation and Projection (UMAP) two-dimensional spatial data visualization, where each point represents a cell, and different colors correspond to different cell types. (2) FeaturePlot: shows the expression of a given gene on an UMAP dimensionality reduction clustered plot. Each point represents a cell, and different colors correspond to different expression (the darker the color, the higher the expression). (3) DotPlot: bubble plots showing the percentage and abundance of gene expression in different cell types. The size of the bubble indicates the percent expression, and the color indicates the abundance of expression. (4) VlnPlot: violin plots showing the expression of a single gene between two or more groups and pathway scores. (5) FeatureScatter: scatter plots for visualizing a possible correlation between two variables.

### Data retrieval and processing

Raw mRNA array data for gastric cancer and clinical data of patients with gastric cancer were retrieved from The Cancer Genome Atlas (TCGA) database (https://portal.gdc.cancer.gov/) in the fragments per kb of transcript per million mapped fragments format. The raw data were processed to remove duplicate samples. We downloaded pan-cancer raw mRNA matrix data and clinical data from the University of California Santa Cruz (UCSC) database (Xena.ucsc.edu/June 2022).

### Sample collection

Tissue samples were collected from patients with gastric cancer who underwent surgical resection at the Second Affiliated Hospital of Harbin Medical University between September 2020 and June 2022. The inclusion criteria were patients aged 18–65 years without endocrine, cardiovascular, hematological, or infectious diseases. The exclusion criteria were pregnant patients, patients with comorbid cancers, and patients who underwent antitumor therapy prior to surgery. All specimens were diagnosed using histopathology by two pathologists based on the diagnostic criteria for gastric cancer. Informed consent was obtained from all patients in writing, and the experiment was approved by the Institutional Review Board (IRB) of the Second Affiliated Hospital of Harbin Medical University (IRB number: KY2021-075).

### Cell culture

GC cells (GES-1, AGS, HGC-27, KATO3, MKN-1, and MKN-45 cell lines) were purchased from Procell Life Science & Technology (Wuhan, China), and were cultured according to the vendor’s instructions. All cell lines were cultured at 37 °C in a 5% CO_2_ humidified chamber (Heal Force, China), and in RPMI-1640 (Gibco, USA) or DME/F12 (HyClone, USA) medium supplemented with 10% fetal bovine serum (FBS) (Gibco, USA) and 1% penicillin/streptomycin (Gibco, USA). Gastric cancer cells were digested with Trypsin (Epizyme, China) during cell passage, and the cells were observed with a 10X microscope (Olympus, Japan) until they were completely exfoliated. The cells were then centrifuged 1000 rpm for 5 min and then resuspended. The concentration of the suspension was calculated by hemocytometer (Marienfeld, Germany), and appropriate cells were taken for further culture.

### Lentiviral transfection

AGS and HGC-27 cells (5 × 10^3^) were cultured in 24-well plates and transfected with previously constructed RNA interference lentiviral vectors (Genechem, China) or a negative control (empty plasmid) for 24 h. The lentiviral interference sequences are shown in Additional file 2: Table S1. The lentivirus was thawed on ice and diluted with complete medium to 1*10^8^ TU/ml, 5*10^7^ TU/ml and 1*10^7^ TU/ml. The experiment was divided into 3 groups according to different culture conditions: M group: 90 µl complete medium + 10 µl lentiviral; A group: 86 µl complete medium + 10 µl lentiviral + 4 µl HitransG A (REVG004, Genechem, China); P group: 86 µl complete medium + 10 µl virus + 4 µl HitransG P (REVG005, Genechem, China). The optimal lentiviral titer and transfection conditions were selected. Twenty-four hours after transfection, the medium was changed to complete medium and gastric cancer cells were cultured for 1 week. The medium was then changed to complete medium containing puromycin (Solarbio, China). After 72 h, the fluorescence intensity was observed under a fluorescence microscope (Olympus, Japan), and the visible fluorescence of the cells indicated that the transfection was successful. The lentivirus was resistant to puromycin, and the stable expression lentiviral cell lines were screened by adding puromycin to the medium. In the process of culture, the cells were overgrown in 24-well plates, and gradually passaged into 12-well plates and 6-well plates.

### Cell countin kit-8 (CCK8) assays

AGS and HGC-27 cells (5 × 10^3^) were seeded into 96-well plates until they were fully adherent to the wall, and five wells were set up for each experimental group. Ten microliters of CCK8 reagent (Cell Counting Kit-8, Beyotime Biotechnology, China) was added to each well and incubated for 2 h. The absorbance was then measured at 450 nm on a microplate reader (Thermo Fisher, USA).

### Colony formation assay

AGS and HGC were seeded in a six-well plate with 1000 cells per plate, and colony formation was visible after 7 days of incubation. An Optical microscope (Olympus, Japan) was used to count more than 50 cell clones counts as a colony. After fixation in 4% paraformaldehyde (Biosharp, China) for 30 min, the cells were stained with 0.5% crystal violet (Solarbio, China) for 30 min.

### 5-Ethynyl-2′-deoxyuridine (EdU) experiment

AGS and HGC (5 × 10^3^) were inoculated into 96-well plates and cultured until the normal growth phase was achieved. Cells were labeled with EdU (Cell-Light EdU Apollo 567 kit, RiboBio, China), fixed, observed, and imaged using a fluorescence microscope (Olympus, Japan).

### Wound-healing assay

AGS and HGC (1 × 10^6^) were cultured in six-well plates until full confluence was achieved and then starved by adding serum-free medium for 24 h. Plates were scratched using a 10 μL pipette tip (Axygen, USA), removing a line of cells. Photographs were taken at 0, 12, and 24 h under an inverted microscope (Olympus, Japan) to observe the degree of wound healing. The scratch area of gastric cancer cells at different time points was calculated using ImageJ software (version 1.8.0).

### Transwell assay

The Transwell assay was performed according to the manufacturer’s instructions. AGS and HGC (2 × 10^4^) were inoculated into a Transwell chamber (Corning, USA) containing 200 µL serum-free medium. The upper chamber surface of the Transwell chamber was coated with Matrigel mix (Corning, USA) to determine the invasion ability of cells. When testing the cell migration ability, the bottom of the chamber was not coated with Matrigel. Medium containing 10% FBS was added to the lower culture plate. After 24 h of incubation, the chamber was removed and stained with crystal violet (Solarbio, China) for 30 min. Five randomly selected fields of view were photographed, and cells were counted under an inverted microscope (Olympus, Japan).

### Western blotting

Sodium dodecyl sulfate polyacrylamide gel electrophoresis (SDS-PAGE, Epizyme, China) was used for western blotting of gastric cancer cells. After lysing cells, the lysate was subjected to electrophoresis, membrane transfer, and blocking of non-specific antigens. This was then incubated overnight at 4 °C with primary antibodies specific for GLUT3, LDHA, L-lactyl, H3K9, H3K18, H3K56, H4K8, and H4K12, N-cad, vimentin,E-cad and Actin (Additional file 3: Table S2). The following day, the membrane was incubated with secondary antibodies for 1 h at room temperature. After visualization of protein bands, grayscale analysis was performed using the ImageJ software (version 1.8.0). The grayscale of the target protein was divided by the grayscale of Actin to obtain the relative amount of the target protein in each protein sample. Then GraphPad Prism 8.0 software was used for statistical analysis of target protein levels between samples.

### Immunohistochemistry

Gastric paracarcinoma and cancerous tissues from patients (n = 7) were immersed in 4% paraformaldehyde (Biosharp, China) overnight, fixed in paraffin, and sectioned at 5 μm. Tissue sections were incubated with antibodies (Additional file 3: Table S2) at 4 °C overnight, followed by incubation with horseradish peroxidase-conjugated secondary antibodies. In addition, we set up a blank control group, that is, PBS (Solarbio, China) was used instead of the primary antibody, and other steps remained unchanged. When the staining result is negative, the staining result is reliable.The samples were then observed and photographed using an optical microscope (Olympus, Japan).

### Immunofluorescence assay

Cells were cultured in 24-well plates. When the cells reached an appropriate density, the medium was aspirated, and the cells were fixed in 1% paraformaldehyde for 15 min, washed twice with PBS, incubated with 3% bovine serum albumin for 30 min, and washed twice with PBS. Specific primary antibodies were added and incubated overnight at 4 °C. Secondary antibodies labeled with Alexa Fluor 488 and 594 (Abcam, USA) were added and incubated for 30 min. Then the cells were washed twice with PBS. DAPI nuclear stain (Solarbio, China) was added and incubated for 10 min.Then the cells were washed twice with PBS. Coverslips (Citotest, China) were removed from the 24-well plates using forceps, inverted, and mounted onto glass slides. Glycerol (Solarbio, China) was added to prevent fluorescence quenching, and the cells were observed and imaged using a confocal microscope (LSM800, Zeiss, Germany).

### Subcutaneous tumor xenograft nude mouse model

Animal experiments were performed in the Animal Experiment Center of the Key Laboratory of Myocardial Ischemia, Second Affiliated Hospital of Harbin Medical University, in strict compliance with the protocol approved by the Institutional Animal Care and Use Committee. Nine 5–7-week-old BALB/c nude mice were purchased from Charles River and housed in a pathogen-free animal facility at 22 °C.Mice had free access to food and autoclaved water. Mice were randomly divided into normal (GLUT3-NC; n = 3), *GLUT3* knockdown (GLUT3-KD; n = 3), and *GLUT3* knockdown + *LDHA* overexpression (GLUT3-KD + LDHA-OE; n = 3) groups. Mice were anesthetized with 2% isoflurane (RWD Life Science, China), and the axillary skin was disinfected using sterile cotton balls. HGC-27 gastric cancer cells were adjusted to a density of 1 × 10^6^/mL, and 100 µL of cell suspension was subcutaneously injected into the axilla using a 1 mL syringe. The tumor volume in each mouse was measured by vernier caliper every 3 days. All mice were sacrificed after 18 days. Tumor tissues were harvested for measurement and weighing, the tumor volume was calculated, and growth curves were plotted. The tumor volume was calculated as volume (mm^3^) = 0.5 × long diameter × short diameter^2^ (mm^2^). Tumors were immersed in 4% paraformaldehyde for 24 h and embedded in paraffin for subsequent immunohistochemical staining.

### Statistics

The survminer R package was used to construct Kaplan–Meier survival plots to estimate the overall survival in the two groups. Data were analyzed and visualized using the SPSS 21.0 and GraphPad Prism 8.0 software, respectively. The Student’s t-test was used to compare means between two groups, and one-way ANOVA was conducted to determine the significance of differences among multiple groups (> 2). P < 0.05 was considered statistically significant.

## Results

### *GLUT3* is highly expressed in gastric cancer and its expression closely relates to lactylation-related pathways

**Fig. 1:**
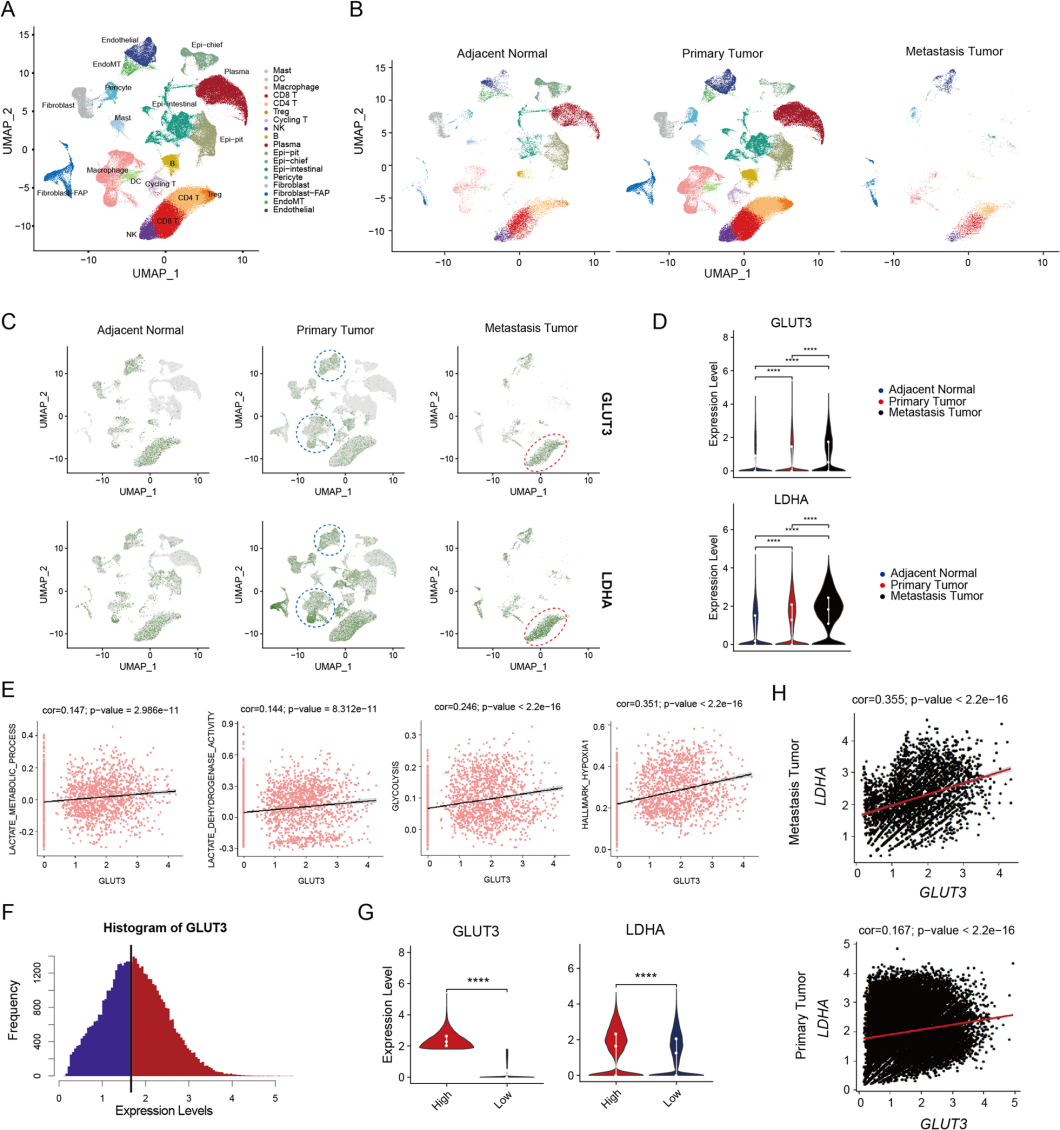
Single-cell sequencing data clusters with cell type annotation. **A** Visualization of all gastric cancer cells using the Uniform Manifold Approximation and Projection (UMAP) function in the R Seurat package (v. 4.0.3). **B** Gastric cancer cells were divided into the tumor-adjacent normal, primary tumor, and metastasis tumor groups according to their origin tissue. **C** UMAP plots showing the distribution and abundance of *GLUT3* and lactate dehydrogenase A (*LDHA*) in gastric cancer. **D** *GLUT3* and *LDHA* expression in tumor-adjacent normal, primary tumor, and metastasis tumor groups quantified using a violin diagram. **E** Scatter plots showing the correlation between the expression of *GLUT3* and lactylation-related pathways. **F** Gastric cancer cells were divided into low and high *GLUT3* expression groups. **G** Violin diagram showing the expression of *GLUT3* and *LDHA* in the low and high *GLUT3* expression groups. **H** Correlation between *GLUT3* and *LDHA* expression in the primary and metastatic tumor groups

Clustering and dimensionality reduction of single-cell data, and cell type annotation resulted in a total of 151,874 entries of high-quality single-cell expression data. The similarities among all cells were calculated through principal component analysis, and the data were visualized in two dimensions. All cells were visualized according to the source of tumor samples and were divided into three categories: tumor-adjacent normal, primary tumor, and metastasis tumor (Fig. 1A, B). Figure 1C shows that GLUT3 and LDHA are highly overlapped in different cells. They were mainly enriched in macrophage and endothelial in the primary tumor group (marked by blue dashed lines), and enriched in NK cells, CD8 T cells, CD4 T cells and Treg cells in metastasis tumor group (marked by red dashed lines). Violin plots were used to quantitatively analyze differences in the expression of *GLUT3* and *LDHA* among the three groups. *GLUT3* and *LDHA* expression levels showed an increasing trend in the tumor-adjacent normal, primary tumor, and metastasis tumor groups (Fig. 1D). In addition, *GLUT3* expression positively correlated with lactylation-related pathways (Fig. 1E). Next, based on *GLUT3* expression, all cells were divided into low and high *GLUT3* groups (Fig. 1F). *LDHA* was also highly expressed in the high *GLUT3* group, indicating that the two genes show the same expression trend (Fig. 1G). Further correlation analysis showed that the expression of these two genes was significantly correlated, with a higher correlation in the metastasis tumor group than in the primary tumor group (Fig. 1H).

### *GLUT3* expression is elevated in gastric cancer and affects prognosis

**Fig. 2:**
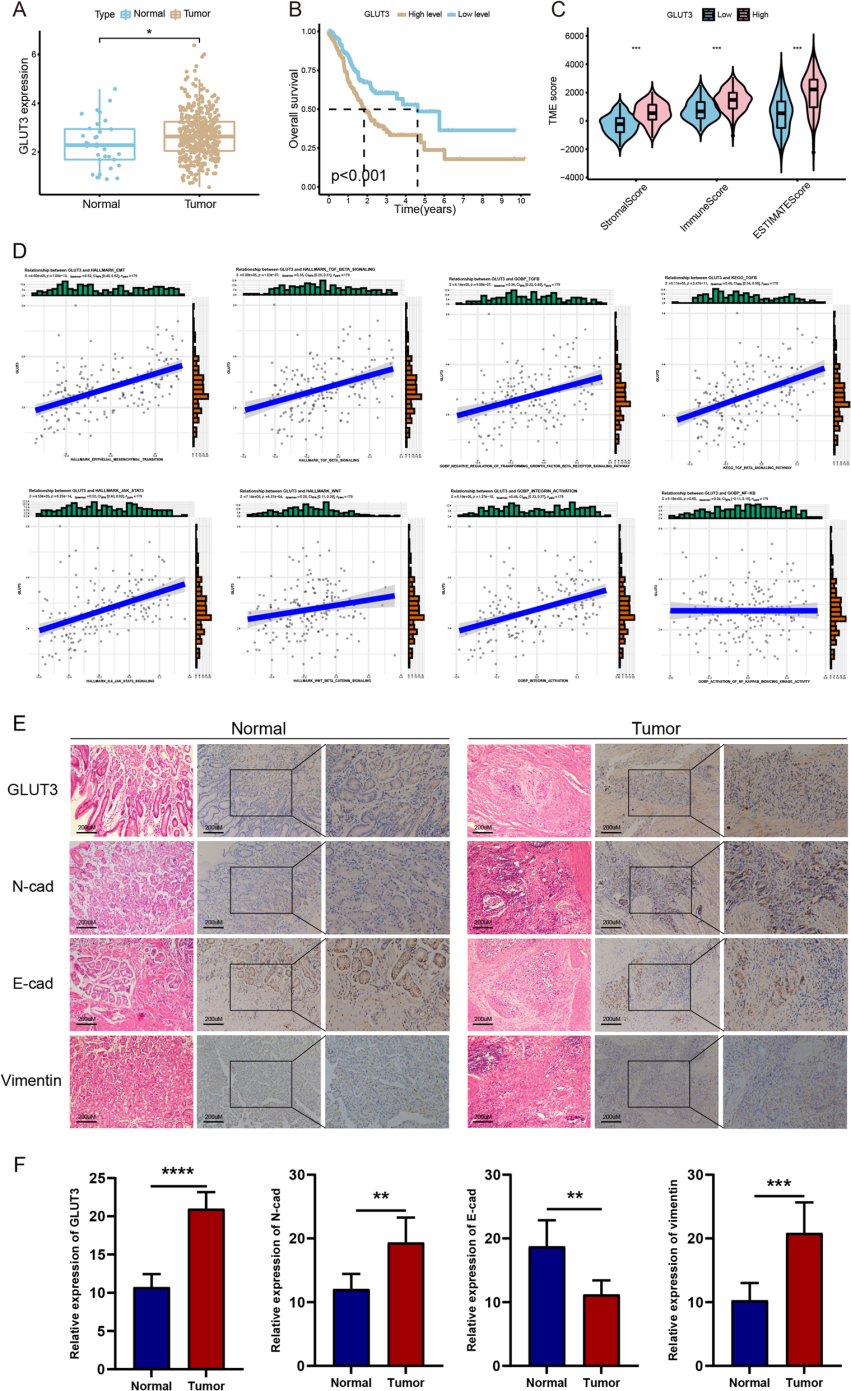
*GLUT3* is highly expressed in gastric cancer and its expression is closely correlated with EMT. **A** *GLUT3* expression in gastric cancer and normal gastric tissues. **B** Classification of samples from the TCGA gastric cancer dataset as high and low *GLUT3* expression groups. Overall survival of patients in the high and low *GLUT3* expression groups. **C** Correlation between *GLUT3* expression and the gastric cancer microenvironment assessed using the ESTIMATE algorithm. **D** Correlation between *GLUT3* expression and the EMT-related regulatory pathway. **E**, **F** Levels of GLUT3, N-cad, E-cad, and vimentin in normal gastric and gastric cancer tissues determined using immunohistochemistry analysis

High-throughput gastric cancer and pan-cancer datasets were downloaded from the TCGA and UCSC databases for epigenetic analysis of *GLUT3*. The expression levels of *GLUT3*were significantly higher in gastric cancer tissues than normal in gastric tissues (Fig. 2A). Overall survival was significantly lower in the high *GLUT3* expression group than in the low *GLUT3* expression group (Fig. 2B). Analysis of the tumor microenvironment between the high and low *GLUT3* expression groups using the ESTIMATE algorithm showed that the stromal score and immune score were significantly higher in the high *GLUT3* expression group than in the low *GLUT3* expression group [17, 18]. This indicated that the proportion of stromal cell and immune cell infiltration was higher in the high *GLUT3* expression group than in the low *GLUT3* expression group. The ESTIMATE score was significantly higher in the high *GLUT3* expression group than in the low *GLUT3* expression group; this indicated that *GLUT3* expression negatively correlated with tumor purity (Fig. 2C). Pan-cancer analysis showed that *GLUT3* expression plays an important role in the development and progression of gastric cancer and a variety of GI tumors. *GLUT3* was highly expressed in a variety of GI tumors (Additional file 1: Figure S1A, B). Moreover, high *GLUT3* expression had a significant effect on the overall and disease-specific survival of patients with GI tumors, but the effect on the disease-free and progression-free survival of these patients was no significant (Additional file 1: Figure S1 C–F). *GLUT3* expression in gastric and colon cancer affected the tumor mutation burden and microsatellite instability levels (Additional file 1: Figure S1G, H). Analysis of *GLUT3* expression in pan-cancer immune cell infiltrates showed that GLUT3 affects immune cell infiltration in various tumors (Additional file 1: Figure S1 I). According to the correlation analysis results, *GLUT3* expression was positively correlated with the HALLMARK_EMT pathway and other classic EMT regulatory pathways (such as TGFβ, JAK_STAT3, WNT, and INTEGRIN_ACTIVITY) [19] (Fig. 2D). Nevertheless, *GLUT3* expression was positively but not significantly correlated with the NF-κB pathway. Immunohistochemistry results showed that GLUT3, N-cad, and vimentin levels were significantly higher in gastric cancer tissues (n = 7) than in normal gastric tissues (n = 7), whereas E-cad levels exhibited the opposite pattern (Fig. 2E, F). The clinical features of the seven patients are shown in Additional file 4: Table S3.

### GLUT3 promotes proliferation, metastasis, and invasiveness of gastric cancer cells through EMT-related pathways

**Fig. 3:**
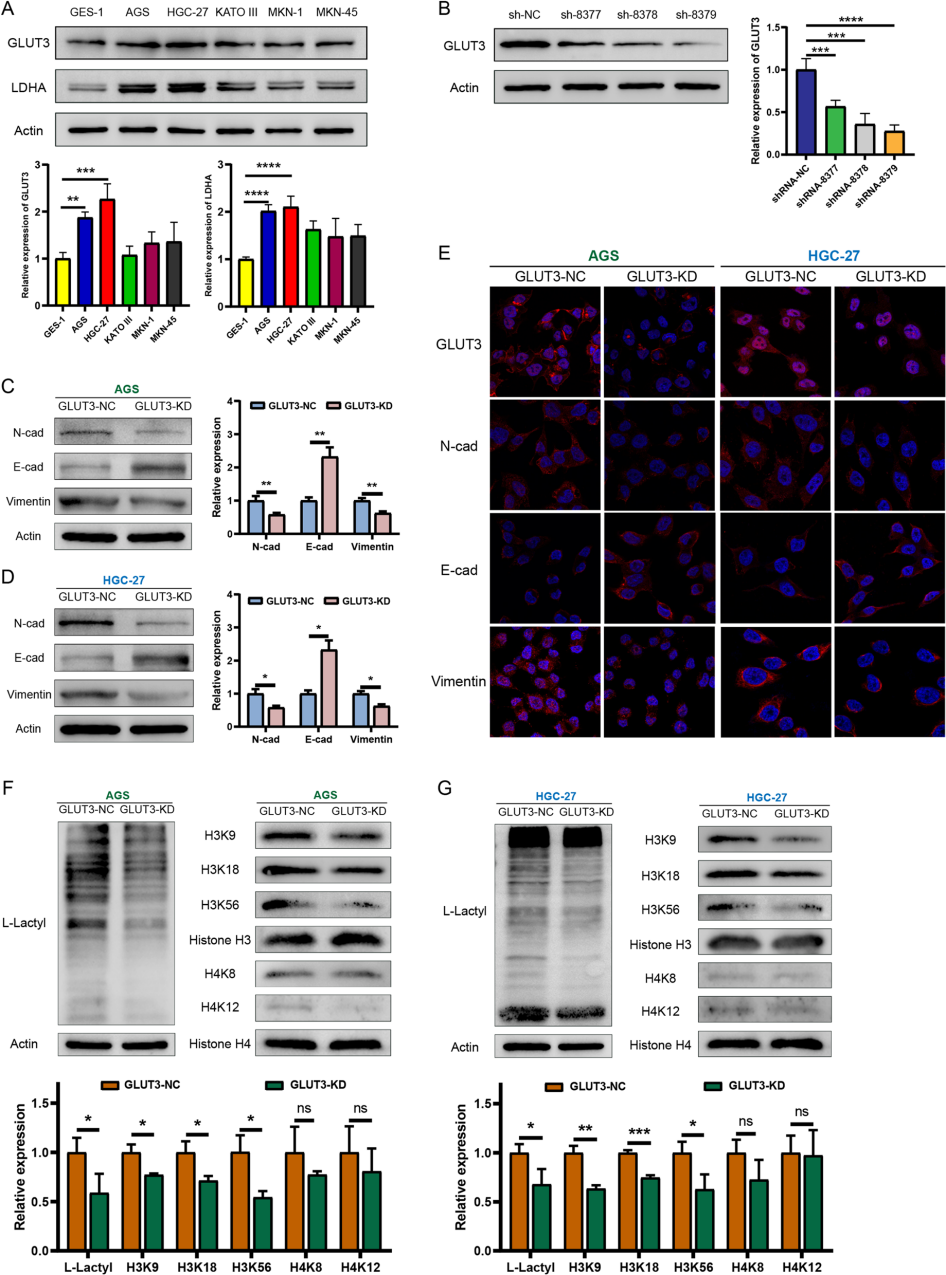
GLUT3 regulates EMT markers and lactylation in gastric cancer. **A** Western blotting of GLUT3 in normal gastric mucosal epithelial cells (GES-1) and gastric cancer cell lines (AGS, HGC-27, KATO III, MKN-1, and MKN-45). **B** Determination of lentiviral *GLUT3* knockdown efficiency in HGC-27 cells. **C**, **D** N-cad, E-cad, and vimentin levels after *GLUT3* knockdown in AGS and HGC-27 cells. **E** GLUT3, N-cad, E-cad, and vimentin levels in AGS and HGC-27 cells after *GLUT3* knockdown detected using immunofluorescence assays. **F**, **G** Overall lactylation and histone H3 lactylation levels in AGS and HGC-27 cell lines after *GLUT3* knockdown

Western blotting showed that GLUT3 and LDHA levels were lower in normal gastric mucosal epithelial cells (GES-1) than in gastric cancer cell lines (AGS, HGC-27, KATO III, MKN-1, and MKN-45) (Fig. 3A). The highest levels were observed in AGS and HGC-27 cells; thus, these cells were selected for subsequent in vitro experiments. A previously constructed *GLUT3* knockdown lentiviral vector was used to transfect HGC-27 cells. The sh-8379 lentivirus was the most efficient knockdown vector (Fig. 3B). *GLUT3* knockdown in AGS and HGC-27 cells resulted in decreased N-cad and vimentin levels, and increased E-cad levels, according to western blot and immunofluorescence results (Fig. 3C–E). Moreover, lactylation in AGS and HGC-27 cells was significantly inhibited by *GLUT3* knockdown (Fig. 3F, G). L-Lactyl is a pan-antibody marker of lactylation that reflects the level of lactylation in tumor samples. L-Lactyl levels were significantly decreased in the GLUT3-KD group, and obvious bands were observed near the 17 kD marker, where the common modification site of histone H3 is found. Therefore, we further investigated whether GLUT3 regulates histone lactylation levels. The levels of lactylation of histone H3 (H3K9, H3K18, and H3K56) in the GLUT3-KD group significantly decreased to different degrees. In addition, although the levels of histone H4 lactylation (H4K8 and H4K12) decreased, these differences were not statistically significant. Therefore, we speculated that GLUT3 affects the functional phenotype of gastric cancer cells mainly by regulating H3 histone lactylation.

**Fig. 4:**
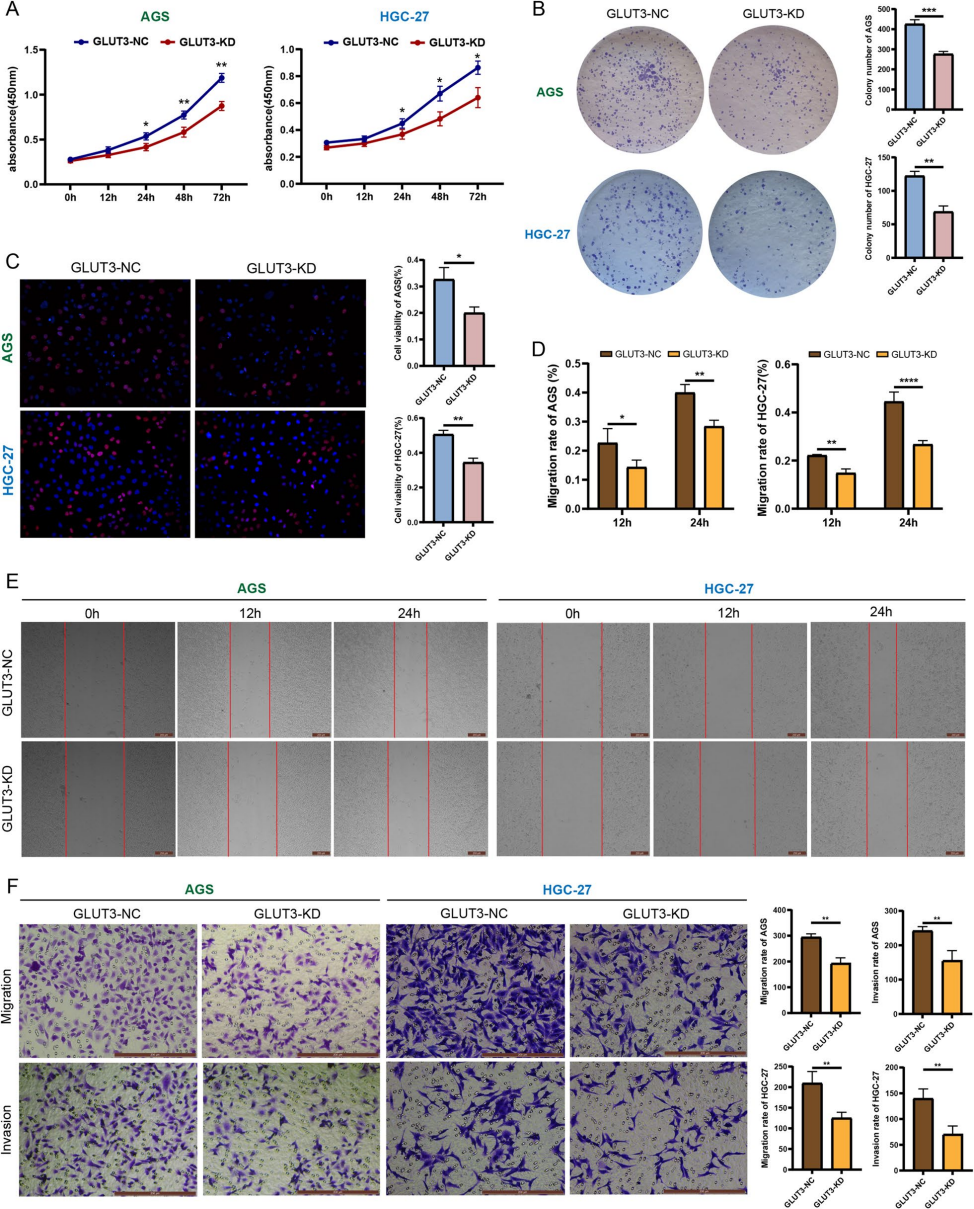
GLUT3 promotes gastric cancer cell proliferation, metastasis, and invasiveness. **A** Cell proliferation capacity in the normal (GLUT3-NC) and *GLUT3* knockdown (GLUT3-KD) groups determined using the CCK8 assay. **B** Cell proliferation capacity in the GLUT3-NC and GLUT3-KD groups determined using the colony formation assay. **C** Cell viability in the GLUT3-NC and GLUT3-KD groups determined using the 5-ethynyl-2′-deoxyuridine assay. **D** Metastatic capacity in the GLUT3-NC and GLUT3-KD groups determined using the scratch assay. **E** Cell metastasis and invasiveness in the GLUT3-NC and GLUT3-KD groups determined using the Transwell assay

CCK8 and colony formation assays showed that the proliferation capacity of AGS and HGC-27 cells was significantly reduced after *GLUT3* knockdown (Fig. 4A , B). EdU assays showed that *GLUT3* knockdown impaired the viability of AGS and HGC-27 cells (Fig. 4C). Scratch assays showed that the scratch width was significantly smaller in the GLUT3-KD group than in the GLUT3-NC group at 12 and 24 h (Fig. 4D, E). Transwell assays showed that the metastatic and invasion capacity was significantly attenuated in GLUT3-KD cells (AGS and HGC-27) compared to GLUT3-NC cells (Fig. 4F). These results suggest that GLUT3 affects EMT and promotes the proliferation, metastasis, and invasiveness of gastric cancer cells.

### LDHA regulates lactylation levels in gastric cancer

**Fig. 5:**
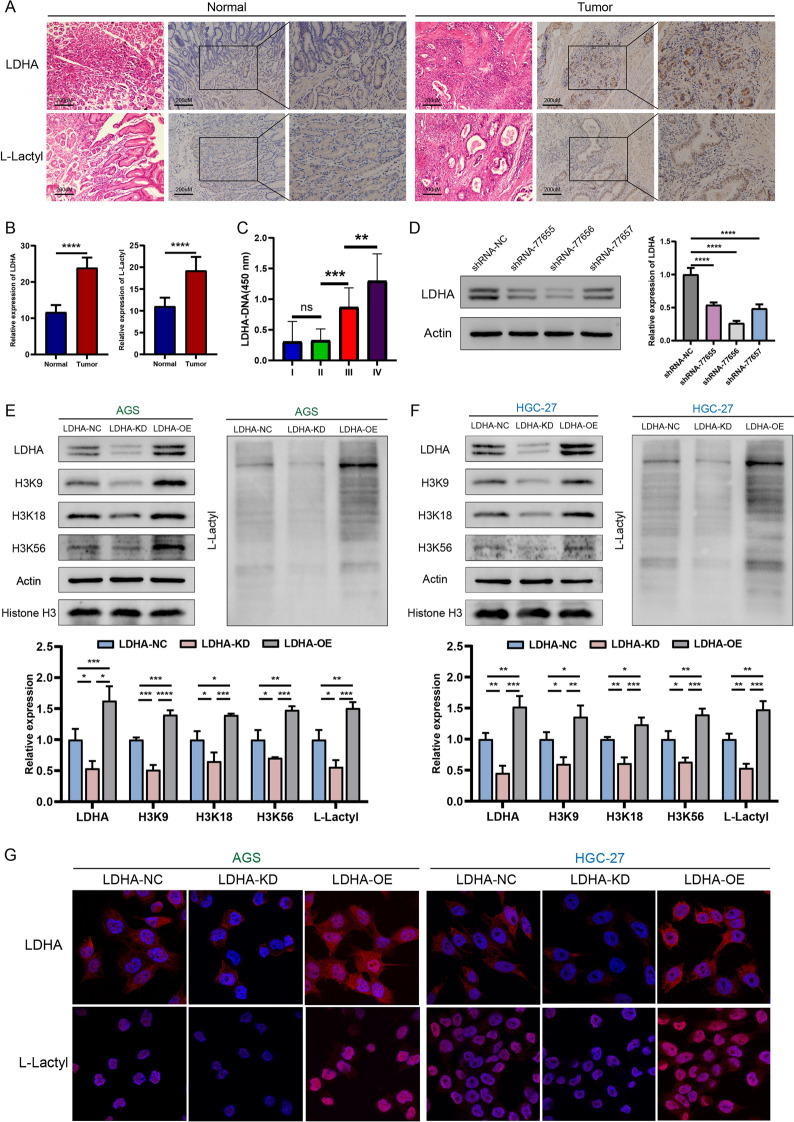
LDHA is highly expressed in gastric cancer and regulates lactylation levels. **A**, **B** LDHA, L-lactyl, H3K9, H3K18, and H3K56 levels in normal gastric and gastric cancer tissues determined using immunohistochemistry. **C** LDHA levels in blood samples from patients at different stages of gastric cancer determined using enzyme-linked immunosorbent assays. **D** Determination of lentiviral *LDHA* knockdown efficiency in HGC-27 cells. **E**, **F** Knockdown and overexpression of *LDHA* in AGS and HGC-27 cells, respectively, and western blot of LDHA, L-lactyl, H3K9, H3K18, and H3K56. **G** LDHA and L-lactyl levels after *LDHA* knockdown and overexpression determined using immunofluorescence assays

Immunohistochemistry results showed that the levels of LDHA and L-lactyl were significantly higher in gastric cancer tissues than in normal gastric tissues (Fig. 5A, B). Blood samples were collected from gastric cancer patients with stage I (n = 15), II (n = 15), III (n = 15) and IV (n = 15) disease for enzyme-linked immunosorbent assays to investigate whether LDHA affects gastric cancer development and progression. The clinicopathological characteristics of the patients are shown in Additional file 5: Table S4. LDHA levels were not significantly different between patients with early-stage gastric cancer (stages I–II) but were significantly elevated in patients with late-stage gastric cancer (stages III–IV) (Fig. 5C). The *LDHA* knockdown shRNA-77656 lentiviral vector was the most efficient for transfection of HGC-27 cells (Fig. 5D). According to western blot analysis, in the LDHA-NC, LDHA-KD, and LDHA-OE groups, the levels of LDHA, L-lactyl, H3K9, H3K18, and H3K56 were significantly decreased and increased after *LDHA* knockdown and overexpression, respectively (Fig. 5E, F). Immunofluorescence results showed that LDHA was primarily expressed in the cytoplasm, while L-lactyl expression was mainly concentrated in the nucleus, with only a small amount in the cytoplasm (Fig. 5G). Therefore, we concluded that LDHA is a key enzyme in lactylation, and that LDHA levels positively correlate with the degree of lactylation.

### GLUT3 enhances gastric cancer cell metastasis and invasiveness by regulating *LDHA* expression

**Fig. 6:**
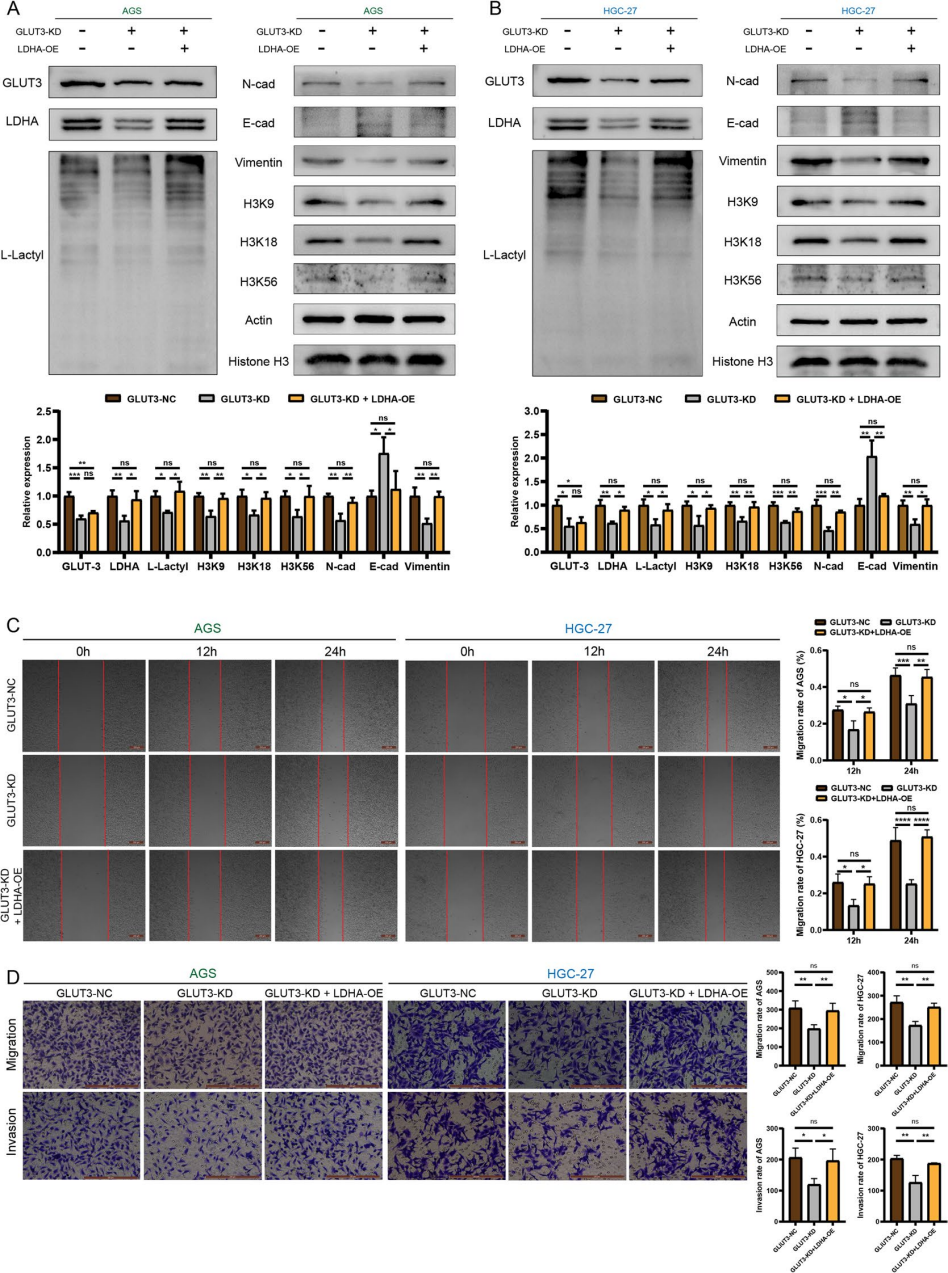
Reversion experiments confirm that GLUT3 promotes gastric cancer cell EMT by regulating *LDHA* expression. **A**, **B** Western blot of GLUT3, LDHA, L-lactyl, H3K9, H3K18, H3K56, N-cad, E-cad, and vimentin in the GLUT3-NC, GLUT3-KD, and *GLUT3* knockdown + *LDHA* overexpression (GLUT3-KD + LDHA-OE) groups. (C) Metastatic capacity of AGS and HGC-27 cells in the GLUT3-NC, GLUT3-KD, and GLUT3-KD + LDHA-OE groups determined using scratch assays. (D) Metastasis and invasiveness of AGS and HGC-27 cells in the GLUT3-NC, GLUT3-KD, and GLUT3-KD + LDHA-OE groups determined using Transwell assays

Reversion assays were performed on gastric cancer cell lines to determine whether GLUT3 regulates EMT through lactylation. The GLUT3-NC, GLUT3-KD, and GLUT3-KD + LDHA-OE groups showed changes in the expression of related genes and the functional phenotypes of metastasis and invasiveness. The levels of GLUT3, LDHA, L-lactyl, H3K9, H3K18, and H3K56 were decreased to different degrees in the GLUT3-KD group (Fig. 6A, B). Additionally, the levels of LDHA, L-lactyl, H3K9, H3K18, and H3K56 were restored, but those of GLUT3 did not significantly increase in the GLUT3-KD + LDHA-OE group. N-cad and vimentin levels were significantly reduced in the GLUT3-KD group, whereas those of E-cad were significantly increased. These changes were reversed to varying degrees in the GLUT3-KD + LDHA-OE group. Moreover, scratch assays showed that the scratch width in the GLUT3-KD group was significantly smaller than that in the GLUT3-NC group, whereas that in the GLUT3-KD + LDHA-OE group was similar to that in the GLUT3-NC group (Fig. 6C). Transwell experiments showed that the migration and invasiveness of GLUT3-KD cells were significantly abrogated, whereas these features were restored in GLUT3-KD + LDHA-OE cells (Fig. 6D). These in vitro results demonstrate that GLUT3 affects gastric cancer cell metastasis and invasiveness by regulating LDHA.

**Fig. 7:**
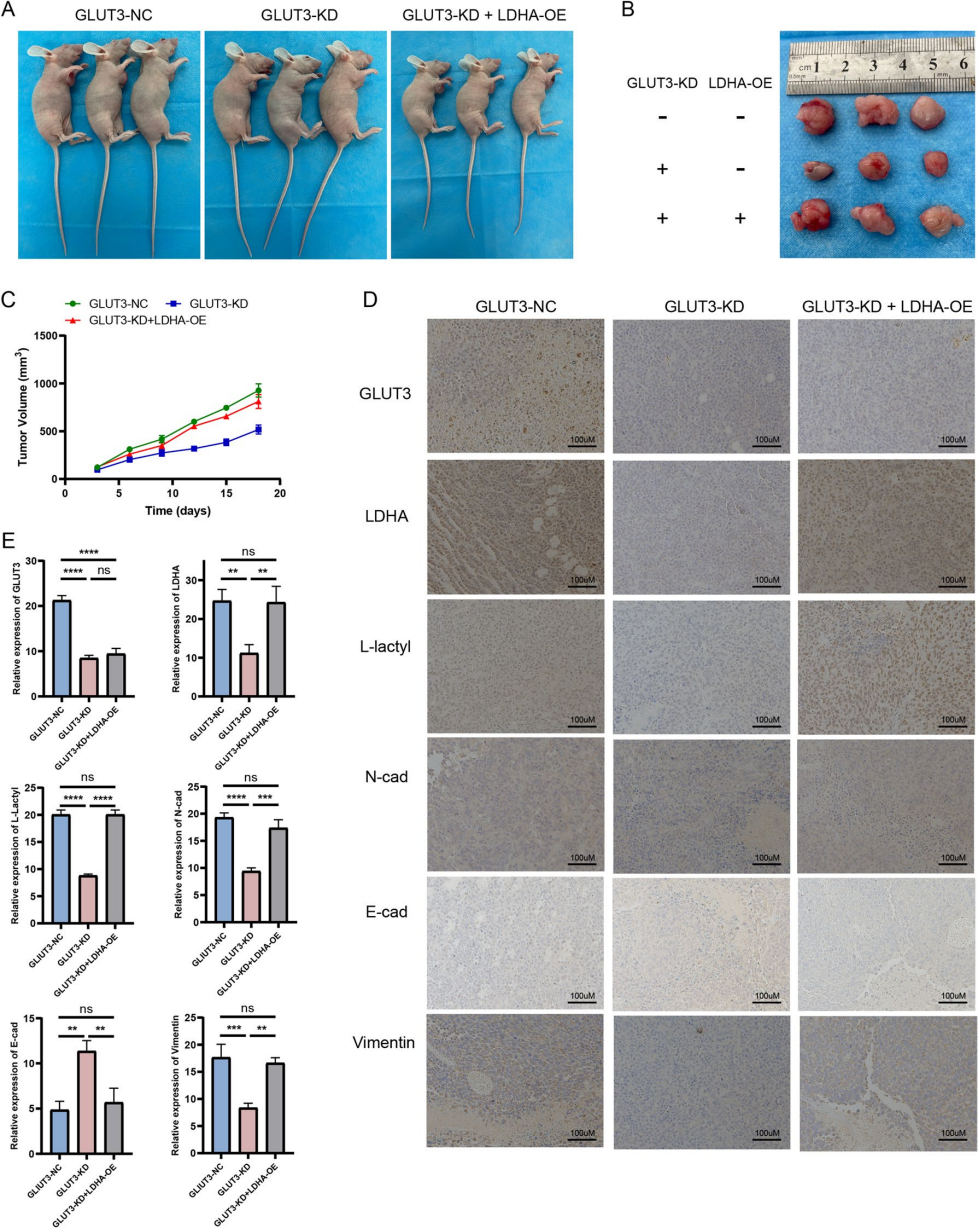
Establishment of a subcutaneous tumor xenograft nude mouse model and expression of target genes. **A** Representative images of tumor-bearing nude mice in the GLUT3-NC, GLUT3-KD, and GLUT3-KD + LDHA-OE groups. **B** Representative images of subcutaneous tumors in the GLUT3-NC, GLUT3-KD, and GLUT3-KD + LDHA-OE groups. **C** Curves of subcutaneous tumor growth in the GLUT3-NC, GLUT3-KD, and GLUT3-KD + LDHA-OE groups. **D** GLUT3, LDHA, L-lactyl, N-cad, E-cad, and vimentin levels in subcutaneous tumors from the GLUT3-NC, GLUT3-KD, and GLUT3-KD + LDHA-OE groups determined using immunohistochemistry assays

Reversion experiments performed on a subcutaneous tumor xenograft nude mouse model (with similar groups as the ones in the in vitro experiments) showed that the tumor size and growth in the GLUT3-KD group were significantly lower than those in the GLUT3-NC group; moreover, the tumor size and growth in the GLUT3-KD + LDHA-OE group did not significantly differ from those in the GLUT3-NC group (Fig. 7A–C). Immunohistochemistry of subcutaneous tumors confirmed that GLUT3 levels were lower in the GLUT3-KD and GLUT3-KD + LDHA-OE groups than in the GLUT3-NC group (Fig. 7D). This indicated that *LDHA* overexpression did not restore *GLUT3* expression after GLUT3 knockdown. Furthermore, the levels of LDHA, L-lactyl, and EMT biomarkers were restored in the GLUT3-KD + LDHA-OE group. Thus, the in vivo and in vitro results were generally consistent.

## Discussion

Glycolysis is one of the ten most significant characteristics of malignant tumors [20]. Several studies have investigated their adverse effects of abnormal glycolysis in tumors; however, few studies have explored the potential of lactate, a metabolite of glycolysis in gastric cancer. Thus, it is currently unknown which biological and biochemical mechanisms GLUT3 and LDHA might interfere with during GC progression and how they correlate to metastasis development. In 2019, a study from The University of Chicago showed that macrophage histones can be lactylated to regulate gene transcription and expression [5]. Furthermore, lactate production and lactylation levels in tumor cells are significantly elevated in a high glucose conditions, indicating a positive correlation between glucose uptake and lactylation levels in these cells. Lactate production and lactylation are reduced after the addition of LDHA inhibitors. This demonstrates that endogenous lactate production is a determinant of histone lactylation levels, and LDHA, a key enzyme in this process, whose expression is positively correlated with lactylation levels. This study explains the epigenetic role of lactate and initiates a wave of research on lactylation. Direct treatment of tumor cells with lactate increases histone lactylation levels and promotes tumor cell proliferation and colony formation [21]. Glycolytic enzymes can undergo lactylation through non-enzymatic reactions, and the level of lysine lactylation positively correlates with glycolytic activity in tissues [22]. In this work, we found that GLUT3 accelerated glycolysis and increased lactic acid production in gastric cancer by regulating LDHA.Lactic acid stored in the tumor microenvironment as a substrate promoted lactylation in gastric cancer cells, ultimately leading to changes in the functional phenotype of tumors.

Lactylation is closely associated with unusual energy metabolism, lactate accumulation, and accelerated glycolysis [23]. Its upregulation is the result of increased glucose consumption by tumor cells. The glycolysis rate of tumor cells is over 200 times higher than normal. Tumor cells must keep high expression of GLUTs to maintain rapid growth, proliferation, and metastasis and to meet the demand for glucose uptake and metabolism[24]. Therefore, overexpression of GLUTs is a major characteristic of tumor cells [25]. For example, GLUT3 is a tissue-specific GLUT with high affinity for glucose, as it is five times higher than that of GLUT1 [26, 27]. Univariate and multifactorial Cox regression analyses indicated that GLUT3 was an independent prognostic factor in patients with multiple tumors [28].Previous reports on GLUT3 have primarily focused on abnormal glucose metabolism and tumor immune microenvironment (TME) [29]. Dai and colleagues found that GLUT3 was highly expressed in colorectal cancer (CRC) and significantly correlated with poor prognosis in CRC patients [11]. However, GLUT1 was not significantly correlated with the prognosis of CRC. Our study also showed that the overall survival rate of patients with high GLUT3 expression was significantly lower than patients with low GLUT3 expression, which indicated that GLUT3 plays an important role in gastric cancer. Furthermore, we selected GLUT3 as an upstream lactylation regulator to prove that GLUT3 expression is upregulated in gastric cancer, leading to an increase in the histone lactylation levels. This ultimately affects the EMT and enhances tumor metastasis and invasiveness in gastric cancer. Lin et al*.* found that GLUT3 promotes colorectal cancer metastasis in high-glucose microenvironments through the YAP/PKM2 pathway [30]. A study from the University of Utah showed that non-small cell lung cancer cells undergoing EMT upregulated GLUT3 expression and increased glucose uptake, thereby promoting tumor cell growth and metastasis [31]. These studies strongly verify that GLUT3 promotes metastasis and invasiveness in various tumors, which supports our results.

As a key substance in cell metabolism, glucose not only provides energy to support cell survival and growth, but also plays an important role in the construction and regulation of TME[32]. Studies have shown that immune cells tend to selectively overexpress GLUT3 for glucose uptake [33]. Additionally, high-throughput glycolysis will eventually forms a tumor microenvironment with high lactic acid and low glucose levels, and immunosuppressive cells, such as myeloid-derived suppressor cells (MDSCs) and regulatory cells (Tregs), adapt well to high lactic acid and low glucose environments [34–36]. Sophia M Hochrein found that GLUT3 is a key gene for the function of T helper 17 (Th17) cells, and GLUT3-dependent histone acetylation can effectively slow the inflammatory process mediated by Th17 cells and is expected to become a new metabolic checkpoint [37]. Related studies suggest that GLUT3 acts as a tumor promoter to accelerate aerobic glycolysis in GC cells. In addition, GLUT3 helps to induce the transition of infiltrating macrophages in the GC microenvironment to the M2 type [38]. Our single cell data analysis also showed GLUT3 and LDHA enrichment in a variety of immune cells, such as Tregs and macrophages, in gastric cancer. Our study also suggests that gastric cancer patients in the high GLUT3 expression group tended to have a higher TMEscore than those in the low expression group, and GLUT3 was also significantly correlated with a variety of immune cells in pan-cancer analysis.

One of the markers of metabolic reprogramming in tumor cells is the upregulation of GLUT1 and GLUT3 [39, 40]. When the tumor metabolic microenvironment is dysfunctional, tumor cells will promote their viability by immune escape. K.J.A found that GLUT family proteins can predict the immunotherapy response of tumor patients through single-cell sequencing and other technology methods. The immunoscore algorithm was used to measure immune cell infiltration in tumors, and the GLUT3/GLUT1 ratio was finally defined as a novel biomarker in the tumor immune microenvironment [41]. With the high expression of GLUT3, glucose metabolism is accelerated, and the levels of lactic acid metabolites are gradually increased. A study from Johns Hopkins University found that lactic acid produced by tumor cells may promote immune escape by passivating CD8 + T cells. Lactic acid accumulation may be central to the immunosuppressive function of the TME and promote tumor growth. By neutralizing the acidic environment in the tumor while maintaining the physiological lactic acid metabolism of cytotoxic CD8 + T cells, the anti-tumor immune response can be enhanced [42]. LDHA associated lactic acid accumulation inhibits immune surveillance of T lymphocytes (T cells) and natural killer (NK) cells by blocking glycolytic flow and interferon-gamma (IFN-γ) production [43]. As our understanding of GLUTs proteins and lactate expands, they may become a potential risk markers and therapeutic targets. Although GLUTs can regulate immune escape and anti-tumor effects, there is no relevant report on whether GLUTs can enhance patients' response to immune checkpoint inhibitors. Recent studies mainly focus on the development of GLUT inhibitors, such as Glutor, which can target GLUT1, GLUT2 and GLUT3 to inhibit glycolytic flow [44]. Princeton University recently reported a variant of GLUT3, called GLUT3exo, that can be used to screen and validate surface inhibitors. This study identified a surface GLUT3 inhibitor, SA47, and elucidated its mode of action through its crystal structure [45]. Elevated levels of LDH are the product of increased glycolytic activity and hypoxia, which can lead to tumor necrosis. Some studies have suggested that the treatment strategy for patients with elevated LDH levels may be to combine immune checkpoint inhibitors and vascular endothelial growth factor (VEGF) inhibitors or tumor reduction therapy [46].

**Fig. 8:**
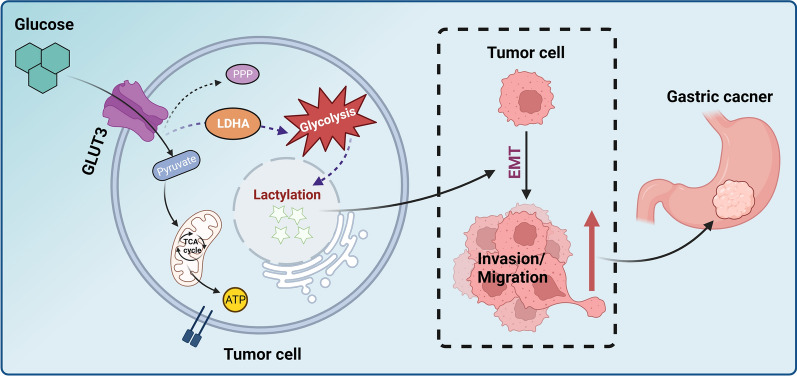
Diagram of the mechanisms underlying glucose transporter (GLUT) 3 regulation of lactylation in gastric cancer. To satisfy the energy needs of tumor proliferation and progression, gastric cancer cells accelerate glucose intake and increase energy metabolism. To that end, they highly express GLUTs to achieve a high glucose oxidation rate, which results in the generation and accumulation of large amounts of lactate. Excess lactate acts as a precursor for histone lactylation, which triggers gastric cancer cell epithelial–mesenchymal transition, and promotes gastric cancer invasiveness and metastasis

Our findings indicated that GLUT3 promotes EMT by regulating lactylation in gastric cancer, eventually resulting in its metastasis and invasiveness. A diagram of the mechanisms revealed by our findings is shown in Fig. 8. These results provide valuable insights into the potential impacts of GLUT3 on glucose metabolism correlates with lactylation. This suggests that tumor metabolic alterations are integrated with epigenetic modifications, and provides a novel direction for subsequent studies [47]. Limitations of this study include the reliance on LDHA and lactylation pan-antibodies to reflect lactylation levels, and the lack of lactylation omics studies or use of antibodies specific to lactylation sites. We aim to investigate this in more depth in the future.

### Supplementary Information

**Additional file 1: Figure S1.** Pan-cancer analysis of GLUT3 expression. **A** GLUT3 expression in 33 cancers. **B** GLUT3 expression in normal and tumor tissues. **C** Overall survival of pan-cancer patients according to GLTU3 expression. **D** Disease-free survival of pan-cancer patients according to GLUT3 expression. **E** Disease-special survival of pan-cancer patients according to GLUT3 expression. **F** Progression-free survival of pan-cancer patients according to GLUT3 expression. **G** Pan-cancer tumor mutation burden according to GLUT3 expression. **H** Pan-cancer microsatellite instability according to GLUT3 expression. **I** Pan-cancer analysis of GLUT3 expression and immune cell infiltration. STAD: gastric cancer, CHOL: bile duct cancer, COAD: colon cancer, PAAD: pancreatic cancer.

**Additional file 2: Table S1.** Interference sequences of lentiviral vectors.

**Additional file 3: Table S2.** Antibody Antibodies used in western blot (WB), immunohistochemistry (IHC) and immunofluorescence (IF) tests.

**Additional file 4: Table S3.** The clinical features of seven patients whose surgical samples were collected.

**Additional file 5: Table S4.** The clinicopathological cahracteristics characteristics of gastric cancer patients whose blood samples were collected.

## Data Availability

All the data and material could be traced from the paper or can be requested from the corresponding author.
