# Supplementary figures and images for: Glucose transporter 3 (GLUT3) promotes lactylation modifications by regulating lactate dehydrogenase A (LDHA) in gastric cancer
Source: Cancer Cell Int. 2023 Dec 1;23:303. doi: 10.1186/s12935-023-03162-8 (PMC10691006; doi:10.1186/s12935-023-03162-8)

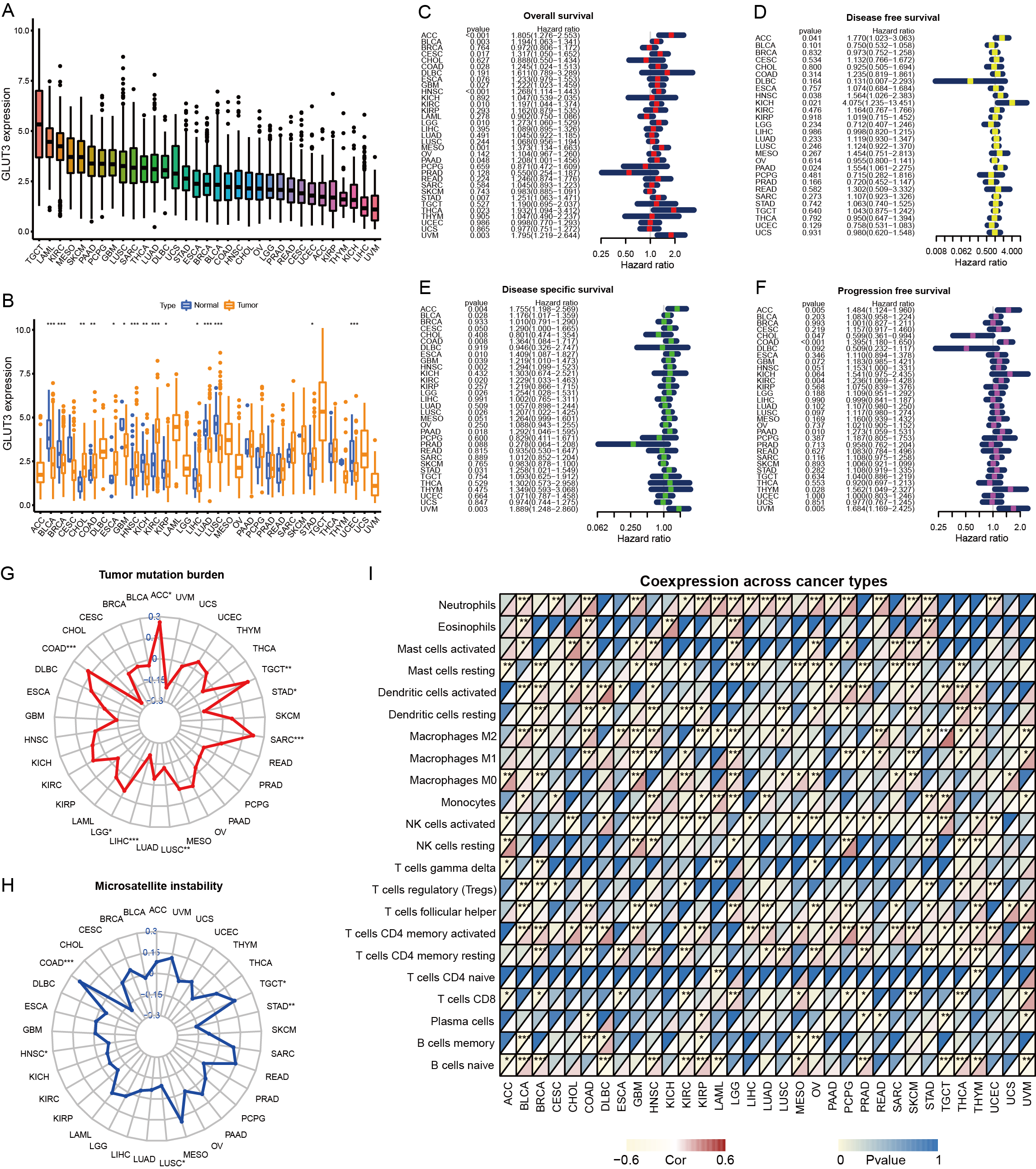

Supplement: Supplementary file 1 — Additional file 1: Figure S1. Pan-cancer analysis of GLUT3 expression. A GLUT3 expression in 33 cancers. B GLUT3 expression in normal and tumor tissues. C Overall survival of pan-cancer patients according to GLTU3 expression. D Disease-free survival of pan-cancer patients according to GLUT3 expression. E Disease-special survival of pan-cancer patients according to GLUT3 expression. F Progression-free survival of pan-cancer patients according to GLUT3 expression. G Pan-cancer tumor mutation burden according to GLUT3 expression. H Pan-cancer microsatellite instability according to GLUT3 expression. I Pan-cancer analysis of GLUT3 expression and immune cell infiltration. STAD: gastric cancer, CHOL: bile duct cancer, COAD: colon cancer, PAAD: pancreatic cancer. [file 12935_2023_3162_MOESM1_ESM.png]
