# Supplementary material for: Glucose transporter 3 (GLUT3) promotes lactylation modifications by regulating lactate dehydrogenase A (LDHA) in gastric cancer
Source: Cancer Cell Int. 2023 Dec 1;23:303. doi: 10.1186/s12935-023-03162-8 (PMC10691006; doi:10.1186/s12935-023-03162-8)
Supplement: Supplementary file 2 — Additional file 2: Table S1. Interference sequences of lentiviral vectors. [file 12935_2023_3162_MOESM2_ESM.docx]

Table S1. Interference sequences of Vector.

| Vector ID | Target sequences information |
| --- | --- |
| SLC2A3-shRNA (8377-1) | AGTAGCTAAGTCGGTTGAAAT |
| SLC2A3-shRNA (8378-1) | CTGGGCATCGTTGTTGGAATT |
| SLC2A3-shRNA (8379-1) | AGAAGAGGAGAATGCTAAGCA |
| LDHA-shRNA (77655-1) | CGAAGACAAATTGAAGGGAGA |
| LDHA-shRNA (77656-1) | GACTGATAAAGATAAGGAACA |
| LDHA-shRNA (77657-1) | ACCTACGTGGCTTGGAAGATA |
| LDHA-oeRNA (82858-3)-p1 | AGGTCGACTCTAGAGGATCCC  GCCACCATGGCAACTCTAAAGG、 |
| LDHA-oeRNA (82858-3)-p2 | ACCGTAAGTTATGTGCTAGCTT  AAAATTGCAGCTCCTTTTGGATC |
