# Supplementary material for: Glucose transporter 3 (GLUT3) promotes lactylation modifications by regulating lactate dehydrogenase A (LDHA) in gastric cancer
Source: Cancer Cell Int. 2023 Dec 1;23:303. doi: 10.1186/s12935-023-03162-8 (PMC10691006; doi:10.1186/s12935-023-03162-8)
Supplement: Supplementary file 3 — Additional file 3: Table S2. Antibody Antibodies used in western blot (WB), immunohistochemistry (IHC) and immunofluorescence (IF) tests. [file 12935_2023_3162_MOESM3_ESM.docx]

Table S2. Antibodies used in western blot (WB), immunohistochemistry (IHC) and immunofluorescence (IF) tests.

| Antibody | Dilution rate | Reference number | Company |
| --- | --- | --- | --- |
| GLUT3 | 1:1000 (WB)  1:100 (IHC)  1:100 (IF) | AF5463 | Affinity |
| N-cad | 1:4000 (WB)  1:1000 (IHC)  1:200 (IF) | 22018-1-AP | Proteintech |
| E-cad | 1:40000 (WB)  1:5000 (IHC)  1:400 (IF) | 20874-1-AP | Proteintech |
| Vimentin | 1:4000 (WB)  1:3000 (IHC)  1:100 (IF) | 10366-1-AP | Proteintech |
| LDHA | 1:5000 (WB)  1:2000 (IHC)  1:100 (IF) | Ab52488 | Abcam |
| L-Lactyl | 1:1000 (WB)  1:100 (IHC)  1:100 (IF) | PTM-1401RM | PTM BIO |
| H3K9 | 1:1000 (WB) | PTM-1419RM | PTM BIO |
| H3K18 | 1:1000 (WB) | PTM-1406RM | PTM BIO |
| H3K56 | 1:1000 (WB) | PTM-1421RM | PTM BIO |
| Histone H3 | 1:1000 (WB) | Ab201456 | Abcam |
| H4K8 | 1:1000 (WB) | PTM-1415RM | PTM BIO |
| H4K12 | 1:1000 (WB) | PTM-1411RM | PTM BIO |
| Histone H4 | 1:1000 (WB) | AF4653 | Affinity |
| Actin | 1:1000 (WB) | TA-09 | ZSGB-BIO |
