# Supplementary material for: Glucose transporter 3 (GLUT3) promotes lactylation modifications by regulating lactate dehydrogenase A (LDHA) in gastric cancer
Source: Cancer Cell Int. 2023 Dec 1;23:303. doi: 10.1186/s12935-023-03162-8 (PMC10691006; doi:10.1186/s12935-023-03162-8)
Supplement: Supplementary file 4 — Additional file 4: Table S3. The clinical features of seven patients whose surgical samples were collected. [file 12935_2023_3162_MOESM4_ESM.docx]

Table S3. The clinical features of seven patients whose surgical samples were collected.

| Sample | Age | Sex | Pathological grade | Histological types | Distant site of metastasis | T | N | M |
| --- | --- | --- | --- | --- | --- | --- | --- | --- |
| patient1 | 61 | F | Ⅲ | Adenocarcinoma | NO | T3 | N2 | M0 |
| patient2 | 57 | F | Ⅱ | Adenocarcinoma | NO | T2 | N1 | M0 |
| patient3 | 55 | M | Ⅲ | Adenocarcinoma | NO | T4 | N1 | M0 |
| patient4 | 49 | F | Ⅱ | Adenocarcinoma | NO | T3 | N2 | M0 |
| patient5 | 45 | M | Ⅱ | Adenocarcinoma | NO | T3 | N0 | M0 |
| patient6 | 63 | M | Ⅲ | Adenocarcinoma | NO | T4 | N2 | M0 |
| patient7 | 58 | M | III | Adenocarcinoma | NO | T3 | N2 | M0 |
