# Supplementary material for: Glucose transporter 3 (GLUT3) promotes lactylation modifications by regulating lactate dehydrogenase A (LDHA) in gastric cancer
Source: Cancer Cell Int. 2023 Dec 1;23:303. doi: 10.1186/s12935-023-03162-8 (PMC10691006; doi:10.1186/s12935-023-03162-8)
Supplement: Supplementary file 5 — Additional file 5: Table S4. The clinicopathological cahracteristics characteristics of gastric cancer patients whose blood samples were collected. [file 12935_2023_3162_MOESM5_ESM.docx]

Table S4. The clinicopathological characteristics of gastric cancer patients whose blood samples were collected.

| Group | Age | Sex | Pathological grade | Histological types | Distant site of metastasis |
| --- | --- | --- | --- | --- | --- |
| blood1 | 58 | M | Ⅰ | Adenocarcinoma | NO |
| blood2 | 65 | F | Ⅲ | Adenocarcinoma | NO |
| blood3 | 47 | M | Ⅰ | Adenocarcinoma | NO |
| blood4 | 28 | M | Ⅳ | Adenocarcinoma | YES |
| blood5 | 49 | M | Ⅰ | Adenocarcinoma | NO |
| blood6 | 65 | M | Ⅰ | Adenocarcinoma | NO |
| blood7 | 54 | M | Ⅲ | Adenocarcinoma | NO |
| blood8 | 52 | M | Ⅰ | Adenocarcinoma | NO |
| blood9 | 54 | F | Ⅲ | Adenocarcinoma | NO |
| blood10 | 57 | F | Ⅲ | Adenocarcinoma | NO |
| blood11 | 60 | M | Ⅱ | Adenocarcinoma | NO |
| blood12 | 54 | F | Ⅱ | Adenocarcinoma | NO |
| blood13 | 56 | F | Ⅲ | Adenocarcinoma | NO |
| blood14 | 51 | M | Ⅱ | Adenocarcinoma | NO |
| blood15 | 62 | F | Ⅲ | Adenocarcinoma | NO |
| blood16 | 61 | F | Ⅰ | Adenocarcinoma | NO |
| blood17 | 39 | M | Ⅲ | Adenocarcinoma | NO |
| blood18 | 62 | F | Ⅱ | Adenocarcinoma | NO |
| blood19 | 49 | M | Ⅳ | Adenocarcinoma | YES |
| blood20 | 52 | M | Ⅰ | Adenocarcinoma | NO |
| blood21 | 47 | M | Ⅳ | Adenocarcinoma | YES |
| blood22 | 48 | M | Ⅲ | Adenocarcinoma | NO |
| blood23 | 53 | M | Ⅱ | Adenocarcinoma | NO |
| blood24 | 57 | M | Ⅰ | Adenocarcinoma | NO |
| blood25 | 53 | M | Ⅱ | Adenocarcinoma | NO |
| blood26 | 53 | F | Ⅲ | Adenocarcinoma | NO |
| blood27 | 39 | M | Ⅱ | Adenocarcinoma | NO |
| blood28 | 64 | M | Ⅳ | Adenocarcinoma | YES |
| blood29 | 51 | F | Ⅱ | Adenocarcinoma | NO |
| blood30 | 48 | M | Ⅰ | Adenocarcinoma | NO |
| blood31 | 51 | F | Ⅲ | Adenocarcinoma | NO |
| blood32 | 65 | F | Ⅲ | Adenocarcinoma | NO |
| blood33 | 40 | F | Ⅱ | Adenocarcinoma | NO |
| blood34 | 55 | M | Ⅰ | Adenocarcinoma | NO |
| blood35 | 53 | F | Ⅲ | Adenocarcinoma | NO |
| blood36 | 65 | F | Ⅲ | Adenocarcinoma | NO |
| blood37 | 63 | F | Ⅱ | Adenocarcinoma | NO |
| blood38 | 59 | M | Ⅲ | Adenocarcinoma | NO |
| blood39 | 48 | M | Ⅰ | Adenocarcinoma | NO |
| blood40 | 61 | M | Ⅰ | Adenocarcinoma | NO |
| blood41 | 55 | F | Ⅲ | Adenocarcinoma | NO |
| blood42 | 54 | F | Ⅳ | Adenocarcinoma | YES |
| blood43 | 62 | F | Ⅳ | Adenocarcinoma | YES |
| blood44 | 60 | F | Ⅱ | Adenocarcinoma | NO |
| blood45 | 58 | F | Ⅱ | Adenocarcinoma | NO |
| blood46 | 50 | M | Ⅳ | Adenocarcinoma | YES |
| blood47 | 53 | M | Ⅰ | Adenocarcinoma | NO |
| blood48 | 61 | F | Ⅳ | Adenocarcinoma | YES |
| blood49 | 55 | M | Ⅱ | Adenocarcinoma | NO |
| blood50 | 57 | F | Ⅳ | Adenocarcinoma | YES |
| blood51 | 54 | M | Ⅰ | Adenocarcinoma | NO |
| blood52 | 63 | F | Ⅳ | Adenocarcinoma | YES |
| blood53 | 47 | F | Ⅳ | Adenocarcinoma | YES |
| blood54 | 63 | F | Ⅱ | Adenocarcinoma | NO |
| blood55 | 46 | M | Ⅳ | Adenocarcinoma | YES |
| blood56 | 56 | F | Ⅳ | Adenocarcinoma | YES |
| blood57 | 45 | M | Ⅰ | Adenocarcinoma | NO |
| blood58 | 51 | F | Ⅳ | Adenocarcinoma | YES |
| blood59 | 44 | M | Ⅱ | Adenocarcinoma | NO |
| blood60 | 49 | F | Ⅳ | Adenocarcinoma | YES |
